# DEBrowser: interactive differential expression analysis and visualization tool for count data

**DOI:** 10.1186/s12864-018-5362-x

**Published:** 2019-01-05

**Authors:** Alper Kucukural, Onur Yukselen, Deniz M. Ozata, Melissa J. Moore, Manuel Garber

**Affiliations:** 1 Bioinformatics Core, University of Massachusetts Medical School, Worcester, MA 01605 USA; 2 Program in Molecular Medicine, University of Massachusetts Medical School, Worcester, MA 01605 USA; 3 RNA Therapeutics Institute, University of Massachusetts Medical School, Worcester, MA 01605 USA

**Keywords:** Differential expression, Data visualization, Interactive data analysis

## Abstract

**Background:**

Sequencing data has become a standard measure of diverse cellular activities. For example, gene expression is accurately measured by RNA sequencing (RNA-Seq) libraries, protein-DNA interactions are captured by chromatin immunoprecipitation sequencing (ChIP-Seq), protein-RNA interactions by crosslinking immunoprecipitation sequencing (CLIP-Seq) or RNA immunoprecipitation (RIP-Seq) sequencing, DNA accessibility by assay for transposase-accessible chromatin (ATAC-Seq), DNase or MNase sequencing libraries. The processing of these sequencing techniques involves library-specific approaches. However, in all cases, once the sequencing libraries are processed, the result is a count table specifying the estimated number of reads originating from each genomic locus. Differential analysis to determine which loci have different cellular activity under different conditions starts with the count table and iterates through a cycle of data assessment, preparation and analysis. Such complex analysis often relies on multiple programs and is therefore a challenge for those without programming skills.

**Results:**

We developed DEBrowser as an R bioconductor project to interactively visualize every step of the differential analysis, without programming. The application provides a rich and interactive web based graphical user interface built on R’s shiny infrastructure. DEBrowser allows users to visualize data with various types of graphs that can be explored further by selecting and re-plotting any desired subset of data. Using the visualization approaches provided, users can determine and correct technical variations such as batch effects and sequencing depth that affect differential analysis. We show DEBrowser’s ease of use by reproducing the analysis of two previously published data sets.

**Conclusions:**

DEBrowser is a flexible, intuitive, web-based analysis platform that enables an iterative and interactive analysis of count data without any requirement of programming knowledge.

**Electronic supplementary material:**

The online version of this article (10.1186/s12864-018-5362-x) contains supplementary material, which is available to authorized users.

## Background

**Fig. 1:**
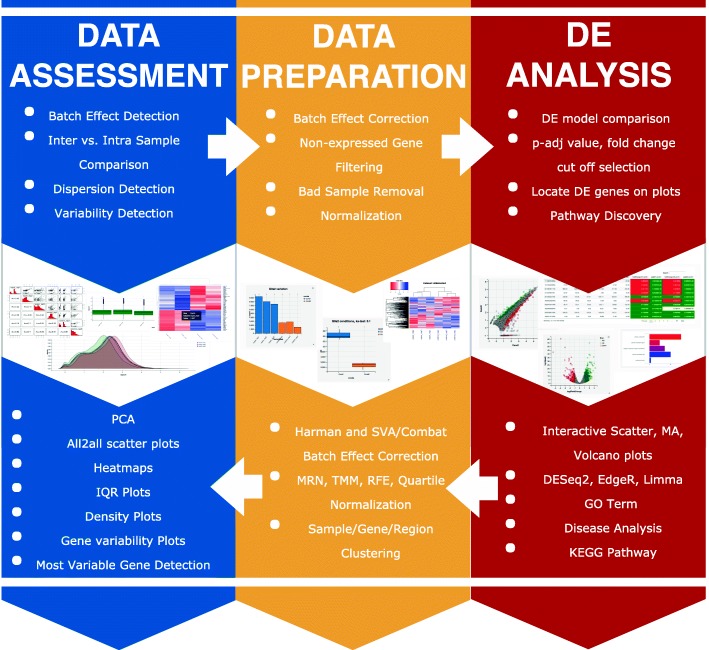
DEBrowser overview

Sequencing techniques have been widely used to measure the activity of genomic regions across conditions. Typical uses include differential expression [1–3], small RNA abundances [4, 5], epigenetic state [6, 7], protein/RNA interactions [8–10] and DNA/RNA interactions [11, 12]. Even though each of these sequencing libraries requires very specific processing steps to determine the genomic loci underlying the observed sequencing reads [13–15], the outputs are always count matrices with rows representing the genomic features of interest such as genes, exons, DNA accessible or DNA bound regions, and the columns being the samples. The values in this table are the estimated number of reads originated from each defined locus for each sample. The most common goal in the analysis of such tables is to find loci exhibiting significant differences between different groups of samples. Differential analysis of count data typically involves an iterative approach that heavily relies on visualization and unsupervised statistical analysis. A typical analysis consists of an iterative application of three main tasks; data assessment, data preparation, and differential expression analysis (Fig. 1). Here we present an application to enable interactive exploration of count matrices obtained after processing of the sequencing output.

Data assessment evaluates the impact of latent factors that may not be related to biological differences. Such differences might come from technical factors, such as DNA/RNA fragmentation, the number of PCR cycles, or sequencing depth that may altogether confound the actual biologically relevant differences present in different samples [16, 17]. Therefore, the count matrix represents a combination of both biological and technical variation. Unsupervised methods such as clustering and principal component analysis (PCA) are powerful ways to identify technical sources of variance [18–20]. Data preparation builds on data assessment to determine and then apply the best approaches to reduce the impact of unwanted sources of variance. Typically this includes the elimination of low quality samples, and filtering or removal of features having low counts [21–25]. Data preparation may also include batch effect correction [26], which removes variability between groups of samples resulting from technical differences in the day, reagents or experimental personnel involved with sample preparation. Following these pre-processing steps often leads to more accurate differential analysis. Differential Analysis seeks to identify features that have higher or lower counts between groups of samples that represent different biological conditions.

We created DEBrowser to enable iterative analysis by non-programmers to achieve similar results to those obtained by investigators well versed in the R programming language. DEBrowser facilitates a modular iterative analysis and visualization process through an intuitive user interface, integrating multiple algorithms and visualization techniques. The goal is to allow users to iteratively inspect and apply each of the many approaches comprised of the three stages described above. DEBrowser provides an evaluation of the results at each analysis step and determines whether further improvements are necessary. DEBrowser goes further than providing static plots or heatmaps: it allows users to explore any anomaly or potential result in an interactive and dynamic manner by zooming-in on data subsets and selecting or hovering over any regions or genes of interest to plot a heatmap, bar, or box plots that updates dynamically based on the user’s selection. For example, users may select the most significantly differentially expressed genes on a volcano plot, and re-display them in a heatmap, that can be further used to inspect the expression of each individual genes across all replicates.

### DE analysis and visualization packages

A number of graphical user interfaces address the need for user-friendly, programming-free visualizations [27–33] (Additional file 1: Table S1). However, all of these approaches have limited interactivity for users to carry out more sophisticated analysis. Similar to DEBrowser, existing tools accept count data as input to visualize, identify, and perform differential analysis and gene ontology. They also visualize results using scatter, MA or volcano plots, as well as heatmaps and PCA plots. DEBrowser goes one step further by enabling hands-on manipulation of the data and by enabling users to re-plot selected subsets of data. These features make DEBrowser a sophisticated tool for data exploration. DEBrowser allows users to color genes that exceed the different fold change cutoffs after differential expression analysis with only a few clicks. Furthermore, all plots are immediately redrawn immediately after changes in the plotting parameters or after any data subsetting operation. Similarly, for easy access to the underlying data, DEBrowser supports hovering to obtain detailed information of individual data points.

In contrast to existing visualization and differential analysis tools, DEBrowser places a strong focus on data assessment and preparation: it intrinsically supports normalization and batch correction methods. Once the user identifies a clear bias in data preparation, processing or sequencing, DEBrowser allows the user to minimize technical variation between samples using proven methods [26, 34–38]. This capability is intended to address the needs of large projects that process samples over an extended period of time, and to help users compare samples available from public repositories originating from different laboratories.

## Implementation

DEBrowser is implemented in R as a shiny application using generic shiny components and layouts [39]. In particular, DEBrowser relies on R’s plotly package [40] both for interactive plots and to display multi-panel data.

Shiny’s reactive programming model is used to update plots upon detection of changes in any input control, plot, or any other bound object. Automatic re-plotting reduces the number of clicks required and therefore improves user experience. When a change in a plot parameter is detected, plots bound to that input are redrawn. As a result, with few clicks, users can, for example, change the highlighted genes from the DE results that exceed a significance of 0.05 and a 2-fold change, to those exceeding a significance of 0.01 and a 10-fold change.

In a typical DEBrowser session users upload a count table obtained by processing of sequencing reads and a table specifying sample information: Conditions and batches. No other inputs are needed (Additional file 2: Figure S1).

## Design and key features

To show the general applicability of DEBrowser on “count data” from different data types; we used a large data set that we recently generated to study gene regulation in innate immune cells (human monocyte derived dendritic cells, hMDDCs) in response to Toll-like receptor signaling [41]. This study generated RNA-Seq, ChIP-Seq and ATAC-Seq [42] to track changes in transcription and regulatory element activity in the course of Toll-like receptor (TLR) signaling. We reprocessed all raw sequence reads either as described in the original publication or using more recent approaches (Additional file 3).

These data are ideal to showcase the main features of DEBrowser and how DEBrowser can be used throughout the analysis cycle. Indeed, we show how DEBrowser was used for data assessment to identify batch effects, and data preparation by filtering low count features and removing batch effects and then performing differential analysis.

### Data assessment

Quality control (QC): Quality control of the count data is a fundamental step in analysis, yet it is not well supported in current applications. With DEBrowser users can easily establish whether normalization, batch correction or sample removal are necessary, or if the data is suitable for differential analysis. To this end, DEBrowser implements PCA, all2all scatter, heatmaps, interquartile range (IQR) and density plots of each sample. These plots can be drawn using a user defined subset of genes, for example by choosing the top N variable genes as defined by coefficient of variance [40, 41]. The subset of genes can be defined graphically by either an expression cutoff or by directly selecting them from another plot.

**Fig. 2:**
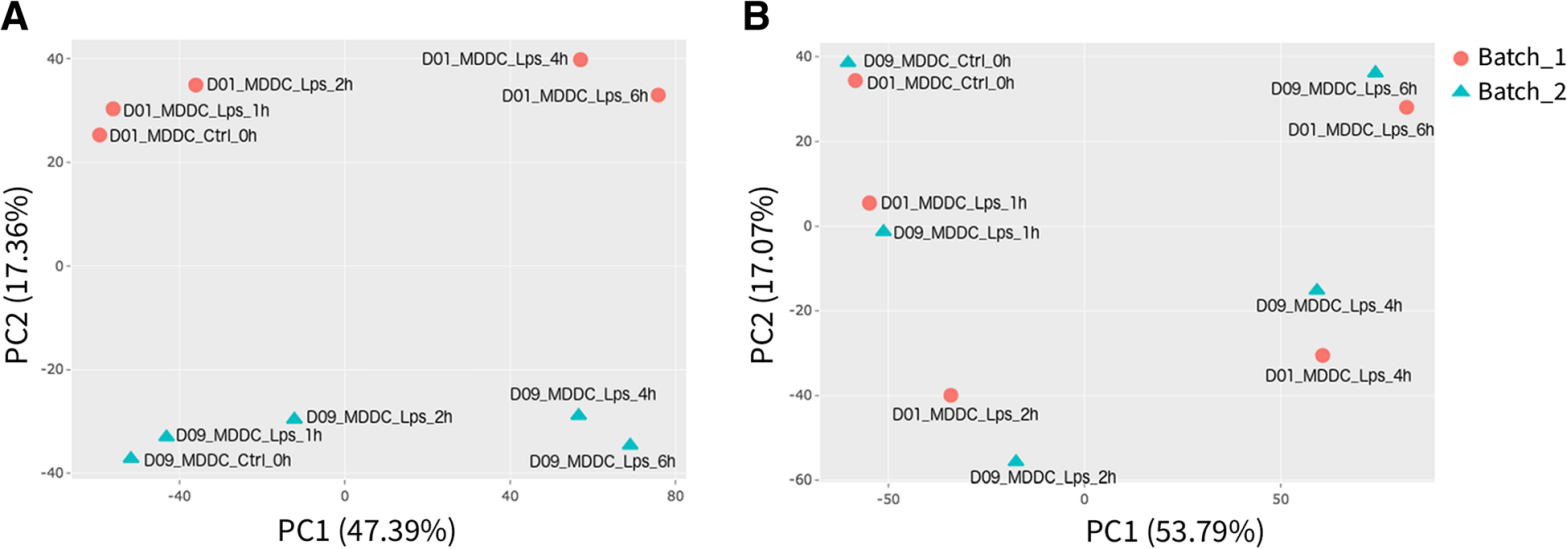
Identifying and correcting batch effects. PCA plot (**a**) before and (**b**) after batch effect correction. Circles and triangles represent batch 1 and 2, respectively

For example, the data for hMDDCs show clear donor dependent differences, which are visible in all2all plots and are captured by the second principal component (Fig. 2a). These differences may be the result of genetic heterogeneity or simply due to technical variability in library construction. Regardless of the source, the variability introduced by inter-donor differences has a direct impact on the power to detect TLR responsive genes.

#### Plots available for data assessment

##### Principal component analysis (PCA)

PCA finds an ordered coordinate transformation whose basis capture, in decreasing order, the most variance in the data. DEBrowser allows users to plot any pair of principal components in a scatter plot. Once users specify sample information (e.g. condition), DEBrowser uses colors or shapes to group samples. These plots are ideal to detect outliers or batch effects (Fig. 2).

##### All2all scatter plots

Gives an overview of sample similarities and variance by plotting all pairwise scatter plots (Additional file 2: Figure S2). Low correlation or high variance across replicates will negatively impact the power to detect DE.

##### Heatmaps

**Fig. 3:**
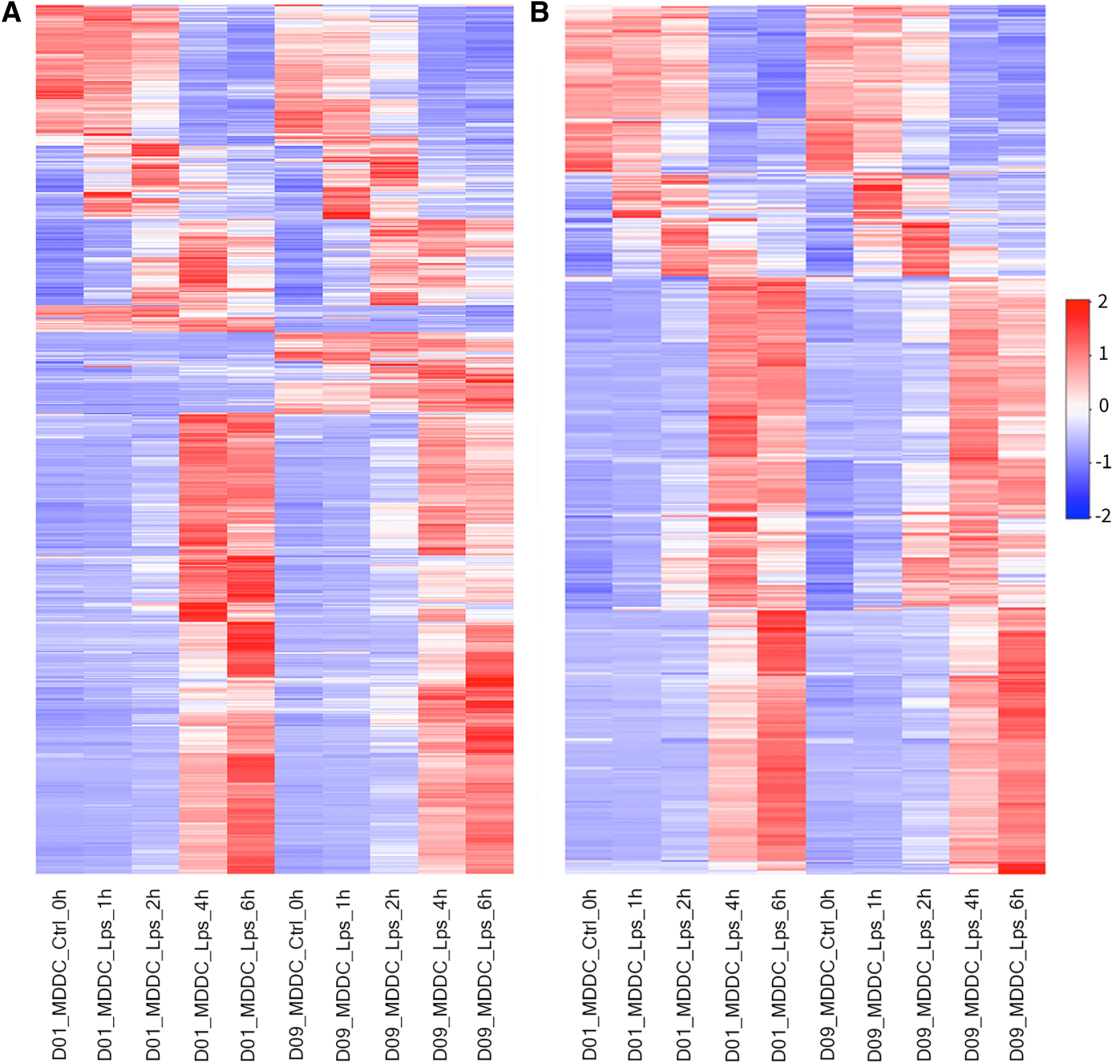
Visualizing the effect of batch correction. Heatmap for normalized RNA-Seq data (log10 transformed, scaled and centered) at different time points from two donors before (**a**) and after (**b**) batch correction. Heatmaps show the top 1000 most varied genes (based on coefficient of variance) whose total minimum counts are higher than 100

DEBrowser allows users select genes based on variance, minimum expression level, DE *p*-value, or after manually selecting a set of genes from any gene centric plots (e.g. scatter, volcano, and other heatmaps). Heatmaps are also useful to assess replicate variability, low quality samples, or batch effects (Fig. 3). Similar to other plots, heatmaps can be used to visualize any type of count data and are ideal to identify global patterns in the data such as dynamic changes in chromatin accessibility following TLR signaling [41] (Additional file 2: Figure S3).

##### IQR and density plots

Interquartile range and density plots display a sample’s quantification distribution in different ways. Using these plots, users can detect any global discrepancy across samples and evaluate the impact of normalization on the distribution of counts. DEBrowser simplifies comparisons by providing plots for both normalized and unnormalized data. Plots are re-drawn as soon as users change the normalization method.

### Data preparation

#### Removal of low coverage features

**Fig. 4:**
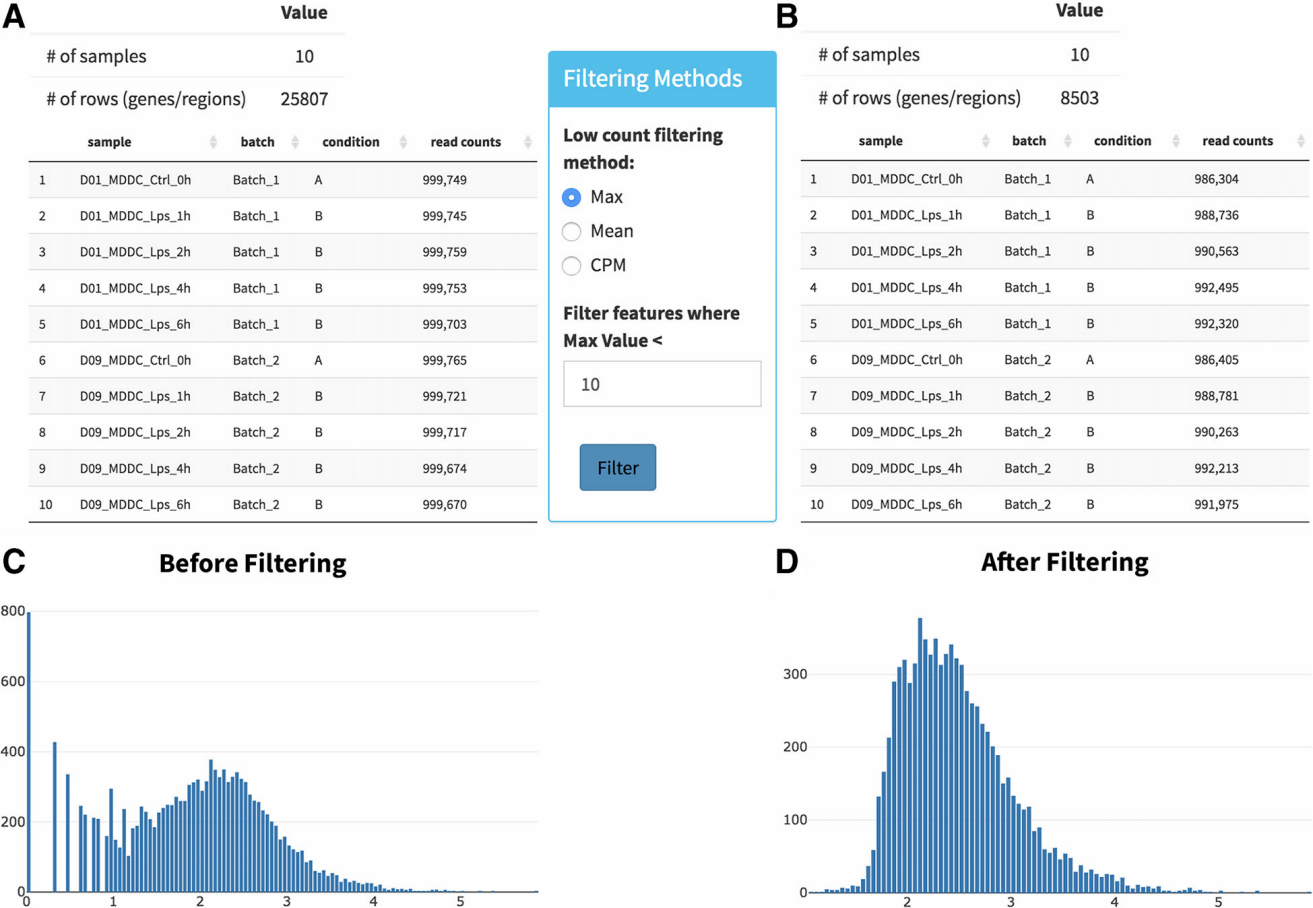
Setting detection levels. DEBrowser displays the total number of counts per library (**a**, **b**) and a histogram of log10 transformed unnormalized feature counts (**c**, **d**) before and after filtering features below the user defined detection level

Removing features (genes, or genomic regions) that have low coverage due to their low expression, increases the speed and accuracy of DE algorithms. It also helps to perform more accurate dispersion calculations and multiple hypothesis correction [43]. DEBrowser provides three common ways to filter these features: by specifying a minimum signal in at least one sample, by a minimum average signal across all samples or by requiring a minimum signal in at least n samples (n defined by the users). Once filtering a criterion is specified, DEBrowser reports read counts for each sample (Fig. 4a, b) and plots the feature count distributions before and after applying the filtering (Fig. 4c, d).

#### Normalization

The count data originating from a sequencing experiment is affected by sequencing depth as well as from differences in the composition of the detected features [44, 45]. DEBrowser supports normalization methods specifically designed for count data: median ratio normalization (MRN) implemented in DESeq2 package [43, 46], Trimmed Mean of M-values (TMM), Relative Log Expression (RLE), and upper quartile methods implemented in the EdgeR package [47]. To evaluate the effect of normalization, DEBrowser immediately displays PCA, IQR and density plots after normalization.

#### Batch effect correction

When quality control shows a clear batch effect that can be traced back to a technical artifact (e.g. different sequencing devices, different personnel, library kits, reagent batch), DEBrowser allows the users, if the experimental design allows batch correction, to minimize the batch effect [48]. The users can specify a batch for every sample via a simple tab separated file that can be created using a text editor or spreadsheet software. Given a batch specification, DEBrowser supports two different methods: ComBat [26, 49] and Harman [50]. The resulting, batch corrected dataset can be evaluated using the same tools available for initial quality control (Figs. 2b, 3b). DEBrowser provides a platform to detect, correct and evaluate the result of batch correction.

### Differential expression (DE) analysis

To demonstrate a typical usage of DEBrowser, we applied DEBrowser on a data set from a previously published study on the role of Jun terminal kinases (JNK1 and JNK2) in the liver and their role in insulin resistance [2]. For this purpose, the authors relied on four different mouse genotypes: wild type (WT), and hepatocyte specific knockouts of Jnk1 and Jnk2 independently (L^Δ1^, L^Δ2^), and a double knockout (L^Δ1,2^). Each genotype was fed either a regular or a high fat diet (HFD). Thereafter, hepatic expression was assayed for each genotype fed with corresponding diet in triplicate using RNA-Seq, resulting in a total of 24 libraries. This study is ideal for DE analysis as it included three replicates per condition. Therefore, we used RSEM [15] for library quantification and DEBrowser to analyze the resulting read count table.

**Fig. 5:**
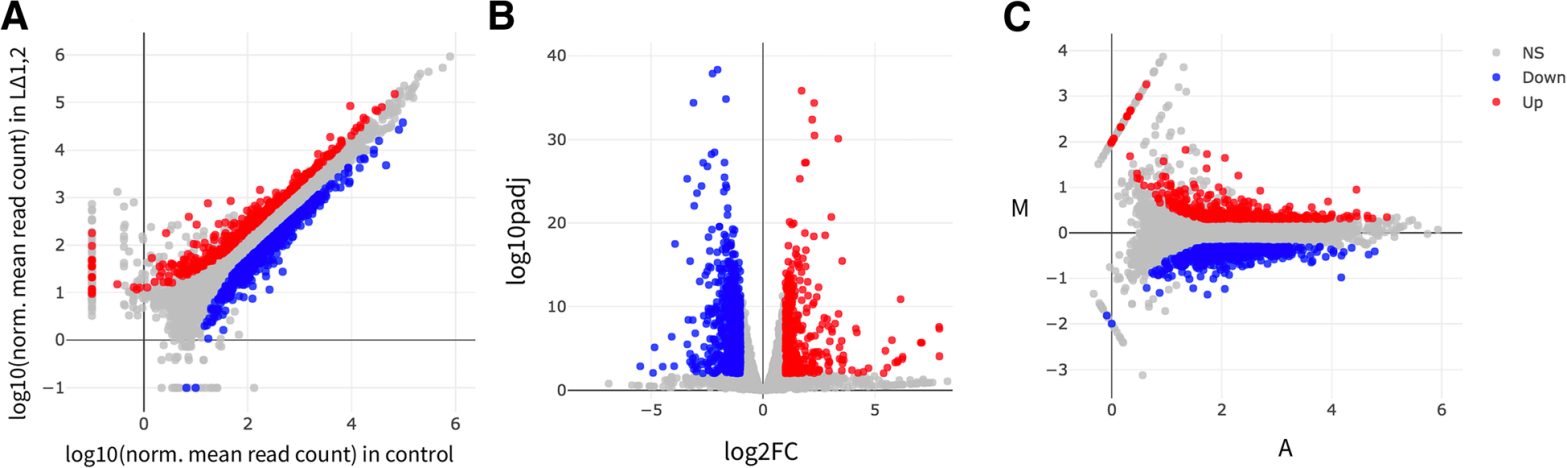
DE visualization. All detected genes are plotted as a (**a**) scatter plot, (**b**) volcano plot, and (**c**) MA plot. Genes that pass a threshold of padj < 0.01 and |log2foldChange| > 1 in differential expression analysis are colored in blue when they are down regulated in control (WT) and red when they are upregulated in the double KO

DEBrowser supports differential analysis using DESeq2 [43], EdgeR [47], and Limma [51]. We used all three methods and present the analysis done with DESeq2 in the main figures and the comparisons between all three methods in the supplementary figures (Additional file 2: Figures S6 and S7). Users can perform differential analysis after defining the groups of samples to compare. DE results can be visualized through the same scatter, volcano and MA plots used for data assessment (Fig. 5a-c). Users can highlight results by specifying desired significance and fold change cut-offs. All plots allow interactive access: Users may select a point within the plot to zoom-in and re-display only selected data. Plots are redrawn as soon as the users change any parameter or select points to zoom-in on any data point or set of points that can be investigated by graphically selecting them.

**Fig. 6:**
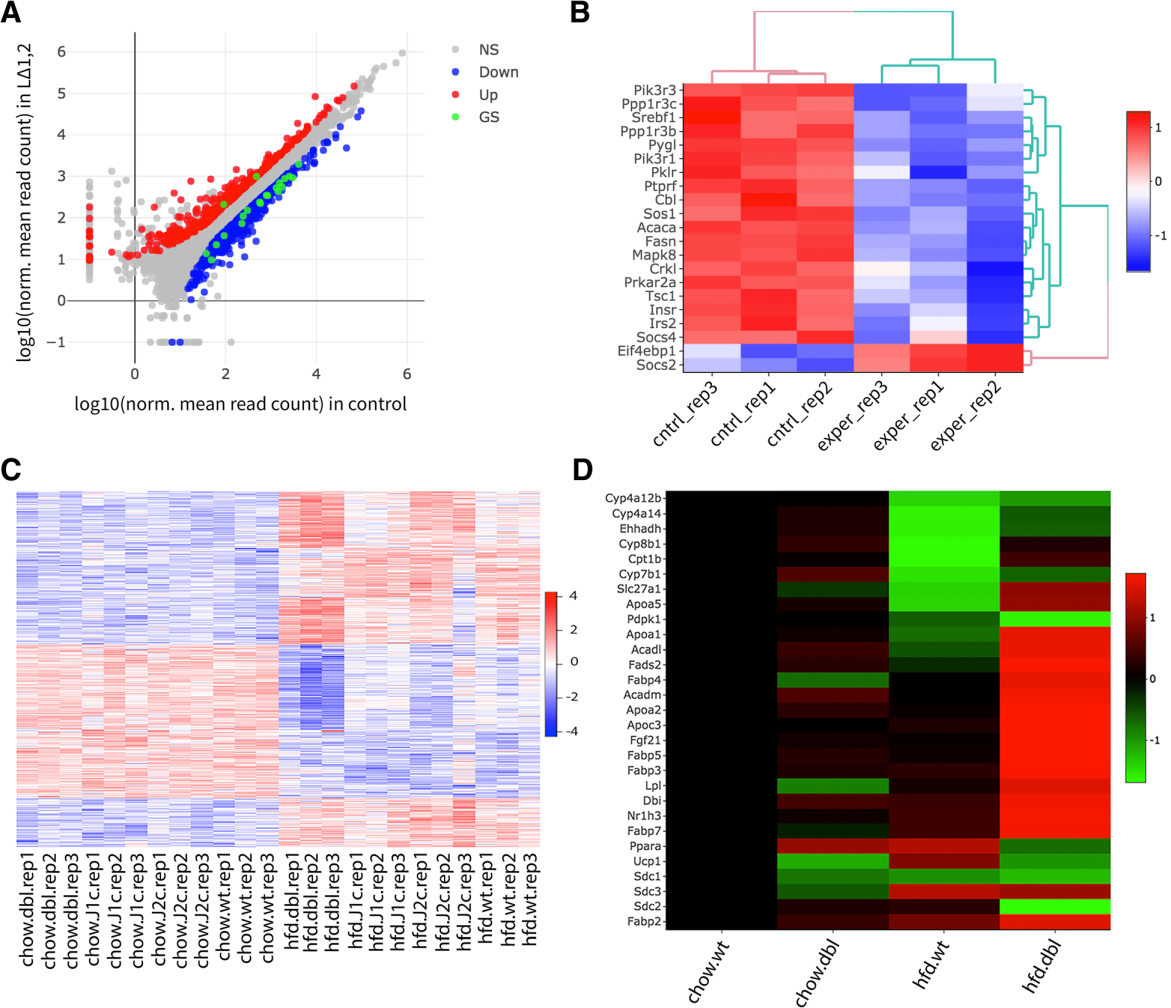
Heat map visualization. Heatmaps of scaled and centered Log_10_ transformed normalized read counts (**a**). As in 5A but with genes annotated in the insulin signaling pathway highlighted in green and (**b**). Only differentially expressed genes involved insulin signaling. **c** All genes differentially expressed in at least one pairwise comparison across all genotypes fed HFD and regular diet (|log2foldChange| > 1 and padj< 0.01). **d** Heatmap showing only genes in the PPARα pathway, averaged across all replicates of a given condition

As reported by the authors, high fat diet has a stronger effect on L^Δ2^ animals compared L^Δ1^ animals (Additional file 2: Figure S8). To examine genes that are dysregulated in the liver under high fat diet fed mice, we performed DE analysis between WT mice fed with a normal or high fat diet. In all, 493 genes are significantly down regulated and 350 are up regulated in livers of mice fed with a high-fat diet (*p* < 0.01, |log2foldChange| > 1). Disease ontology analysis of up regulated genes shows, not surprisingly, a clear enrichment of diseases resulting from poor diet (urinary, kidney and other obesity related ailments). Here to show an example, we overlaid enriched insulin signaling pathway genes on a scatter plot (Fig. 6a) and easily created a heatmap (Fig. 6b) by using selected genes on this scatter plot similar to that in Additional file 2: Figure S3-B in the original report [2].

We then compared the effect of both normal and HFD on all four genotypes. To do so we performed pairwise comparisons between all conditions and selected all genes with a |log2foldChange| > 1 and padj< 0.01 in at least one comparison to display in a heatmap similar to Additional file 2: Figure S3-A in the original report (Fig. 6c). Finally, we used DEBrowser to reproduce Additional file 2: Figure S3-B in original report. To do so we performed gene ontology analysis of the DE genes between WT and L^Δ1,2^ on HFD, selected genes that are annotated as part of the PPARα pathway and visualized them as a heatmap (Fig. 6d).

It is important to note that the original publication used much older DE methods [52]. When we applied DESeq2 to replicate the analysis we found that it had much greater power to detect differentially expressed genes and indeed at a similar threshold many more genes are called DE. Nevertheless, there is a very good agreement (73%) in the calls made by both methods (Additional file 2: Figure S4), and most importantly, there are no differences with the Gene Ontology enrichments reported in the original publication (Additional file 2: Figure S5).

**Fig. 7:**
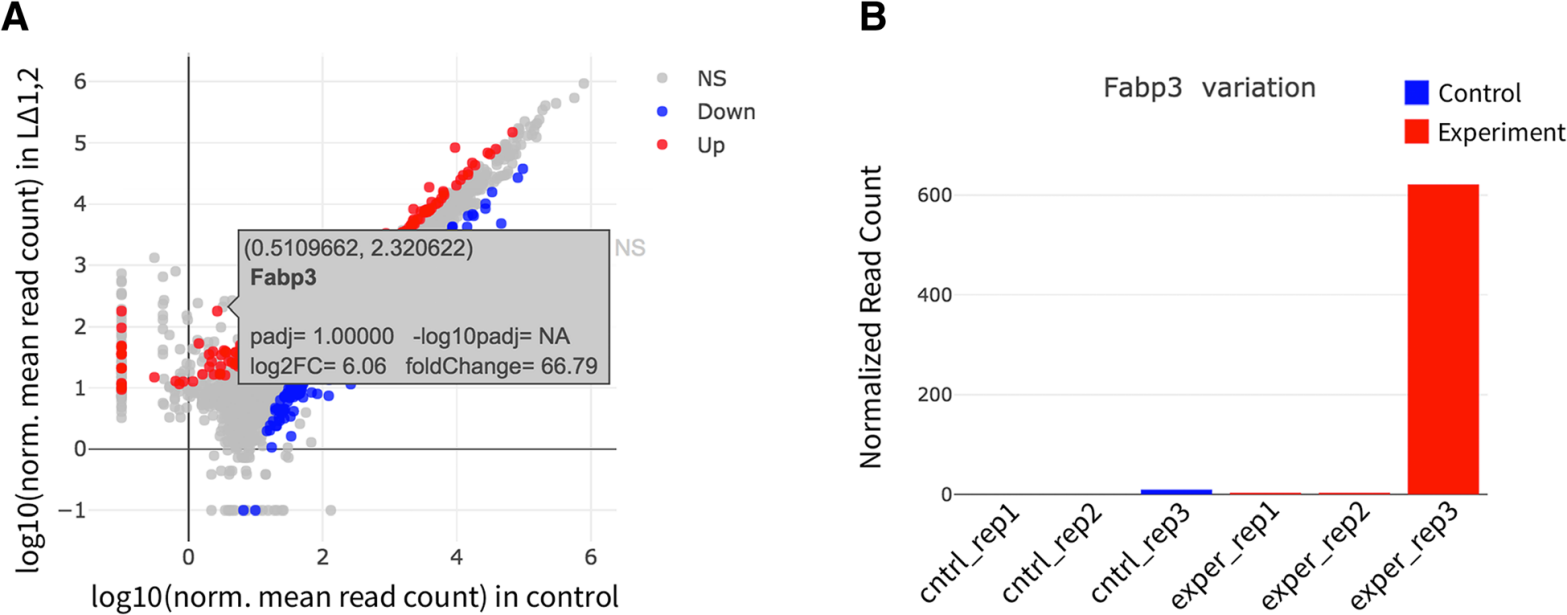
Why are genes with high fold change not significantly DE? **a** FABP3 gene is hovered over on a scatter plot. **b** The read counts for FABP3 gene is shown as a bar plot, showing high intra condition variability which explains the lack of significance in DE

Further, users can explore individual regions by hovering over points. The gene or region id is shown and a bar plot displaying the values of the gene or region across all samples is drawn. This is especially useful to investigate, for example, why certain genes may have large differences in between samples but fail to achieve statistical significance. In Fig. 7a, the, Fabp3 gene fails to achieve a significance of 0.05 despite of its mean expression between the conditions exceeding a 50-fold change. Hovering over Fabp3 shows the high variance of this gene across samples, which explains why statistical tests that account for both inter and intra condition variability fail to achieve significance in cases of high intra condition variability (Fig. 7b).

**Fig. 8:**
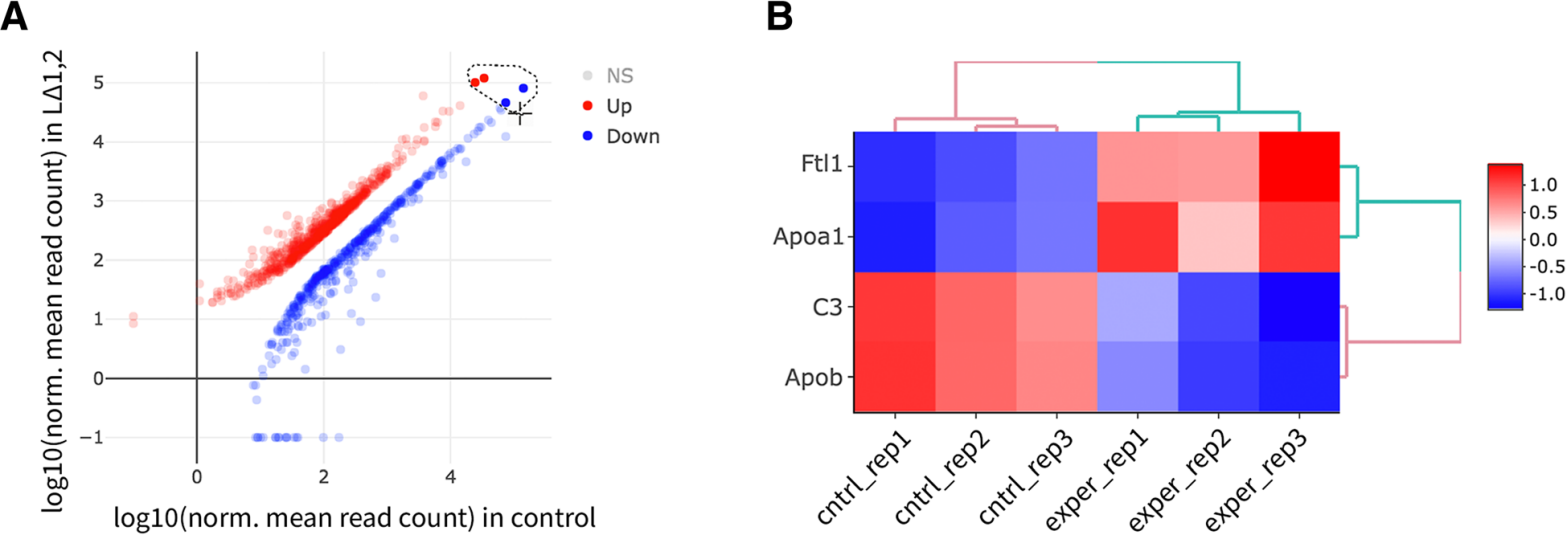
Gene selection using free form drawing. **a** Scatter plot of up and down regulated genes. **b** Heatmap of lasso selected of two up (Apob, C3) and two down regulated genes (Apoa1, Ftl1)

Volcano, Scatter and MA plots work on summary statistics: significance averages or fold change of averages. To explore the underlying data for any set of regions in a plot, DEBrowser can draw heatmaps for any selected region from any main plot. Selection can be made in a rectangular form or as a free-form using plotly’s lasso select (Fig. 8a), which then dynamically generates a heatmap of the selection. (Fig. 8b). Conversely, in any heatmap the users can select a subset of regions (such as based on similar expression pattern) for downstream analysis such as gene ontology, disease and pathway analysis.

### Gene ontology, disease and pathway discovery

**Fig. 9:**
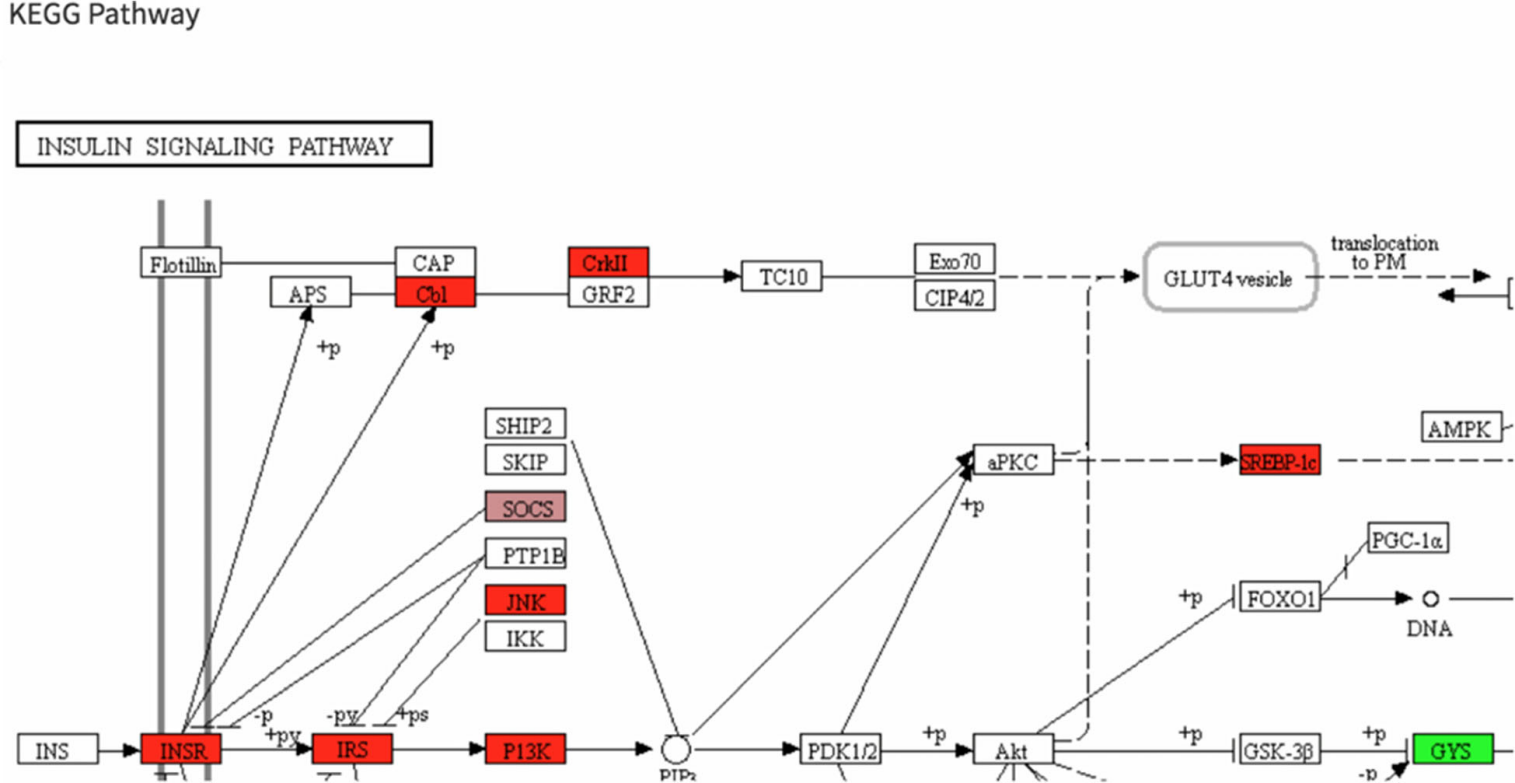
Pathway visualization. Insulin signaling pathway. Detected DE genes shown with colors according to their fold changes

For gene expression analysis in particular, DEBrowser supports Gene Ontology (GO) [53], KEGG pathway [53] and disease ontology analysis [54]. Users can perform GO or Pathway analysis directly on the results of differential expression analysis or on a subset of selected genes from any of the plots described above. For KEGG pathway analysis, in particular, DEBrowser provides pathway diagram for each enriched category (Fig. 9).

**Fig. 10:**
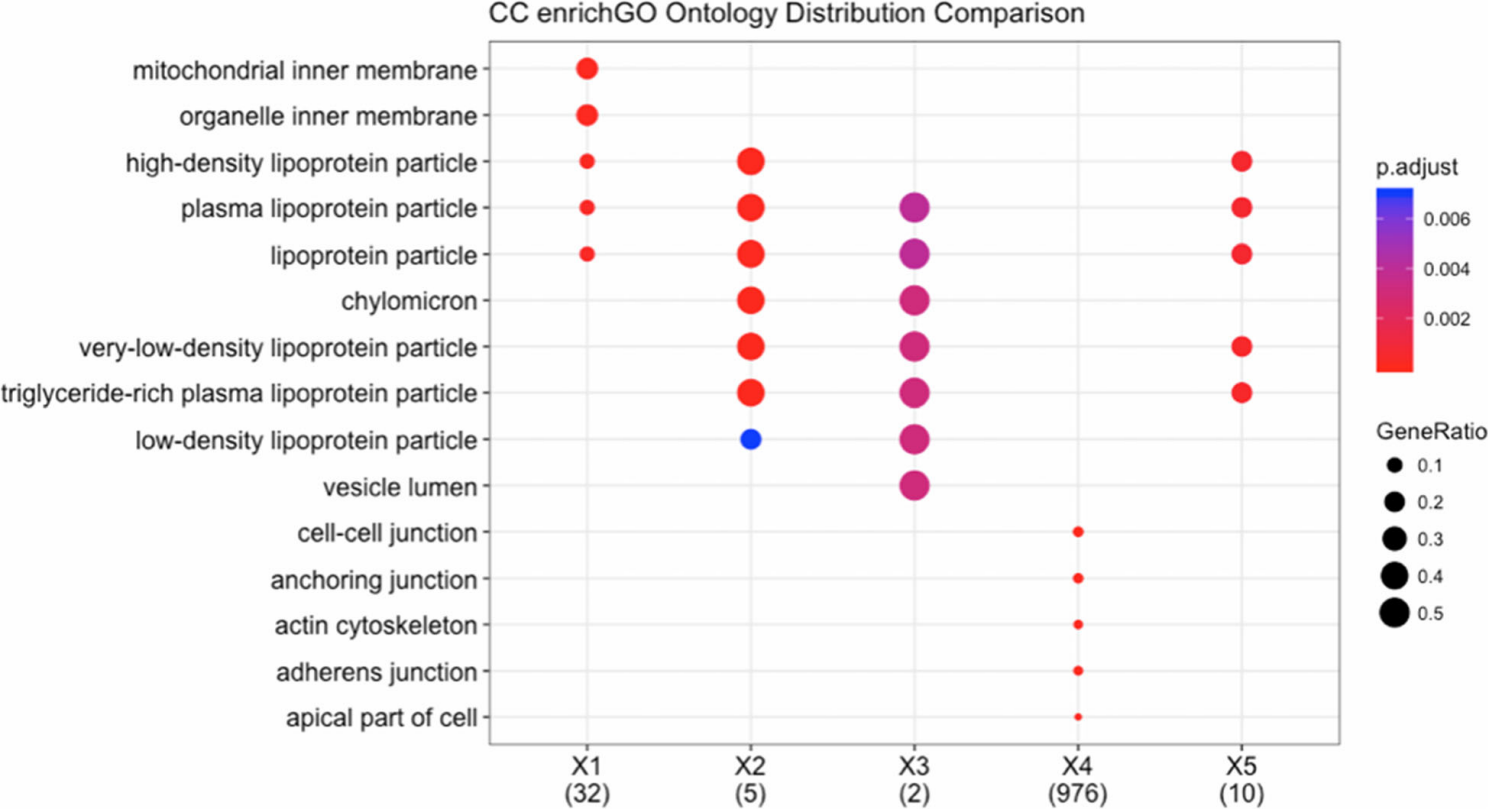
Ontology comparisons in subclusters. An example of a cluster profiler plot

To further assist users in differential analysis, DEBrowser provides k-means clustering of differential regions, and when these regions are associated with genes, a gene ontology enrichment analysis is performed using enrichGO function in ClusterProfiler package [53] (Fig. 10).

### Comparison to related applications

There are several applications, with varying functionalities, available for the exploration and analysis of DE. Most notable ones are, OASIS [27], VisRseq [28], DEGUST [29], DEIVA [30], WebMeV [31], Chipster [32], and DEapp [33]. A comparison of DEBrowser features to those applications is shown in Additional file 1: Table S1.

### DEBrowser modular design

To reduce the code complexity and manage the program easier the components were designed in a modular fashion, so that while DEBrowser grows larger, it is easy to build on top of the simple modules. To this end, bar, box and scatter plots, heatmaps modules could be reused multiple times in DEBrowser. We also shared example shiny applications that use individual modules. This modularity increased our development, test speed, and code reusability. For example, the size and the margins of the plots are controlled within the same module in all the plots in DEBrowser. This modular design allows other users to repurpose any of the tools built into DEBrowser for their own packages.

## Conclusion

Existing tools do not fully support the full process of differential expression analysis and visualization. Additionally, the plots are usually static and do not allow interactivity to understand the different parts of the data using different parameters reducing the efficiency of data exploration.

In contrast, the DEBrowser application provides users, who do not have any programming experience, the ability to perform their own analysis in an iterative and interactive process that responds dynamically to user inputs. DEBrowser leverages open source components that are in active development in bioconductor [55, 56], thus it benefits from a large community of developers. Its modular design makes it easy to swap components shall new paradigms or projects emerge that provide more ideal functionality than currently available. Therefore, it fills a much-needed void in graphical user interfaces for the analysis of count data that is typical of sequencing assays.

## Additional files

Additional file 1: Application feature comparison table. (DOCX 24 kb)

Additional file 2: Supplementary figures. (DOCX 4395 kb)

Additional file 3: Data processing methods and installation instructions. (DOCX 18 kb)

## Abbreviations

ATAC-Seq: Assay for transposase-accessible chromatin
ChIP-Seq: Chromatin immunoprecipitation sequencing
CLIP-Seq: Cross-linking immunoprecipitation sequencing
GO: Gene Ontology
hMDDCs: Human monocyte derived mouse dendritic cells
IQR: Interquartile range
JNK1 and JNK1: Jun terminal kinases
L^Δ1^: JNK1 knockout
L^Δ1,2^: JNK1 and JNK2 double knockout
L^Δ2^: JNK2 knockout
MRN: Median ratio normalization
PCA: Principal component analysis
QC: Quality control
RIP-Seq: RNA immunoprecipitation sequencing
RLE: Relative Log Expression
RNA-Seq: RNA sequencing
TLR: Toll-like receptor
TMM: Trimmed Mean of M-values
WT: Wild type
