# Supplementary material for: DEBrowser: interactive differential expression analysis and visualization tool for count data
Source: BMC Genomics. 2019 Jan 5;20:6. doi: 10.1186/s12864-018-5362-x (PMC6321710; doi:10.1186/s12864-018-5362-x)
Supplement: Supplementary file 1 — Application feature comparison table. (DOCX 24 kb) [file 12864_2018_5362_MOESM1_ESM.docx]

Table S1. Application feature comparison table.

|  | DEBrowser | OASIS | VisRseq | DEGUST | DEIVA | WebMeV | Chipster | DEapp |
| --- | --- | --- | --- | --- | --- | --- | --- | --- |
| Batch-effect | yes | no | no | no | no | no | no | no |
| Perform DESeq2 | yes | no | no | no | no | yes | yes | yes |
| Perform EdgeR | yes | no | no | no | no | yes | yes | yes |
| Perform Limma | yes | no | no | no | no | yes | no | yes |
| locate genes | yes | yes | no | yes | yes | no | no | no |
| Identify differential expression | yes | yes | yes | yes | yes | yes | yes | yes |
| Scatter Plot | yes | no | yes | no | no | no | yes | no |
| MA-plot | yes | no | yes | yes | yes | no | yes | no |
| Volcano plot | yes | yes | yes | yes | yes | no | yes | yes |
| all2all scatter | yes | no | no | no | no | no | no | no |
| PCA | yes | no | no | no | no | yes | yes | yes |
| heatmap | yes | yes | yes | yes | no | yes | yes | no |
| interactive heatmap | yes | no | no | no | no | no | no | no |
| Standalone heatmap | yes | no | no | no | no | no | no | no |
| Density histogram | yes | no | no | no | no | yes | no | no |
| IQR plots | yes | no | no | no | no | no | no | yes |
| GO Term Analysis | yes | no | no | no | no | yes | no | no |
| Disease | yes | no | no | no | no | no | no | no |
| KEGG Pathway Analysis | yes | no | no | no | yes | yes | no | no |
| Complex DE comparisons | yes | no | no | no | no | no | no | Only models |
| users data | yes | no | yes | yes | yes | yes | yes | yes |
| license | GPL v3 | LGPLv2 | NA | GPL v3 | MIT | GPL v3 | GPL v3 | GPL v2 |
| browser | yes | yes | NA | yes | yes | yes | yes | yes |
| development | R | NA | Java, R | bash, node.jsb | node.js | Java, R | Java | R |
| Modular structure | yes | no | no | no | no | no | no | no |

- **Batch-effect**: can apply batch effect correction on user dataset.
- **Locate genes**: includes functionality to visually locate the position of the features/genes.
- **Identify differential expression: can identify differentially expressed genes/features and mark them in various plots.**
- **MA-plot**: can render the DGE statistical test result as a MA-plot (i.e. a scatter plot of mean expression vs log fold change).
- **Volcano plot**: can render the DGE statistical test result as a volcano plot (p-value vs fold change).
- all2all scatter
- **Interactive heatmap**: includes a functionality to select a group of features to zoom in/out on the plot and visualize the DGE statistical test results of each block.
- **Standalone heatmap: heatmap module can run as a standalone application.**
- **Density histogram: can render histograms of counts per gene**
- **IQR plots: shows interquartile range for dataset**
- **GO Term Analysis:** can perform enrichment analysis on gene sets
- **KEGG Pathway Analysis:** can perform analysis on Kyoto Encylopedia of Genes and Genomes (KEGG) pathway database
- **Disease: can perform Disease Ontology (DO) analysis.**
- **Users data**: the user can visualize their own datasets.
- **Browser**: the application opens in a browser.
- **Development:** software developed in these languages**.**
- **Modular structure**: design allows using modules in other applications.
