# Supplementary material for: DEBrowser: interactive differential expression analysis and visualization tool for count data
Source: BMC Genomics. 2019 Jan 5;20:6. doi: 10.1186/s12864-018-5362-x (PMC6321710; doi:10.1186/s12864-018-5362-x)
Supplement: Supplementary file 2 — Supplementary figures. (DOCX 4395 kb) [file 12864_2018_5362_MOESM2_ESM.docx]

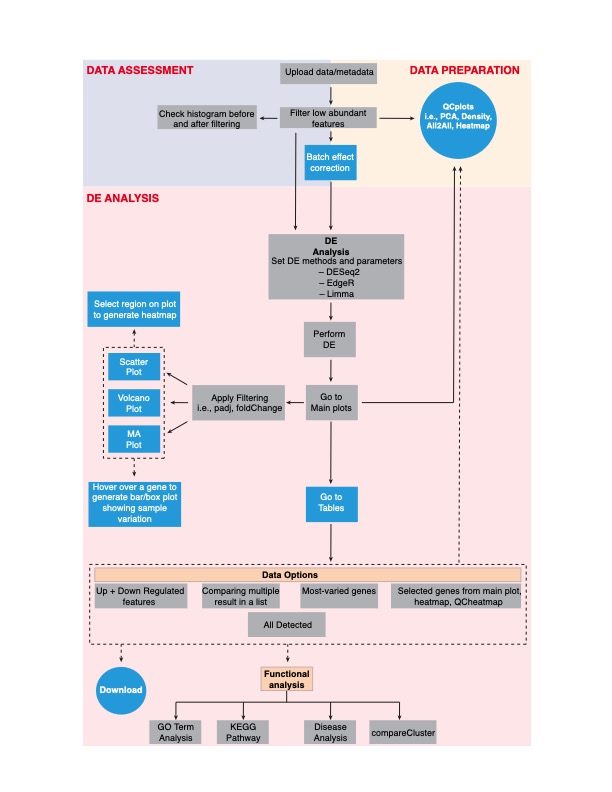


**Figure S1.** General workflow diagram for DEBrowser. The normalization methods can be applied in the steps shown with the blue boxes.


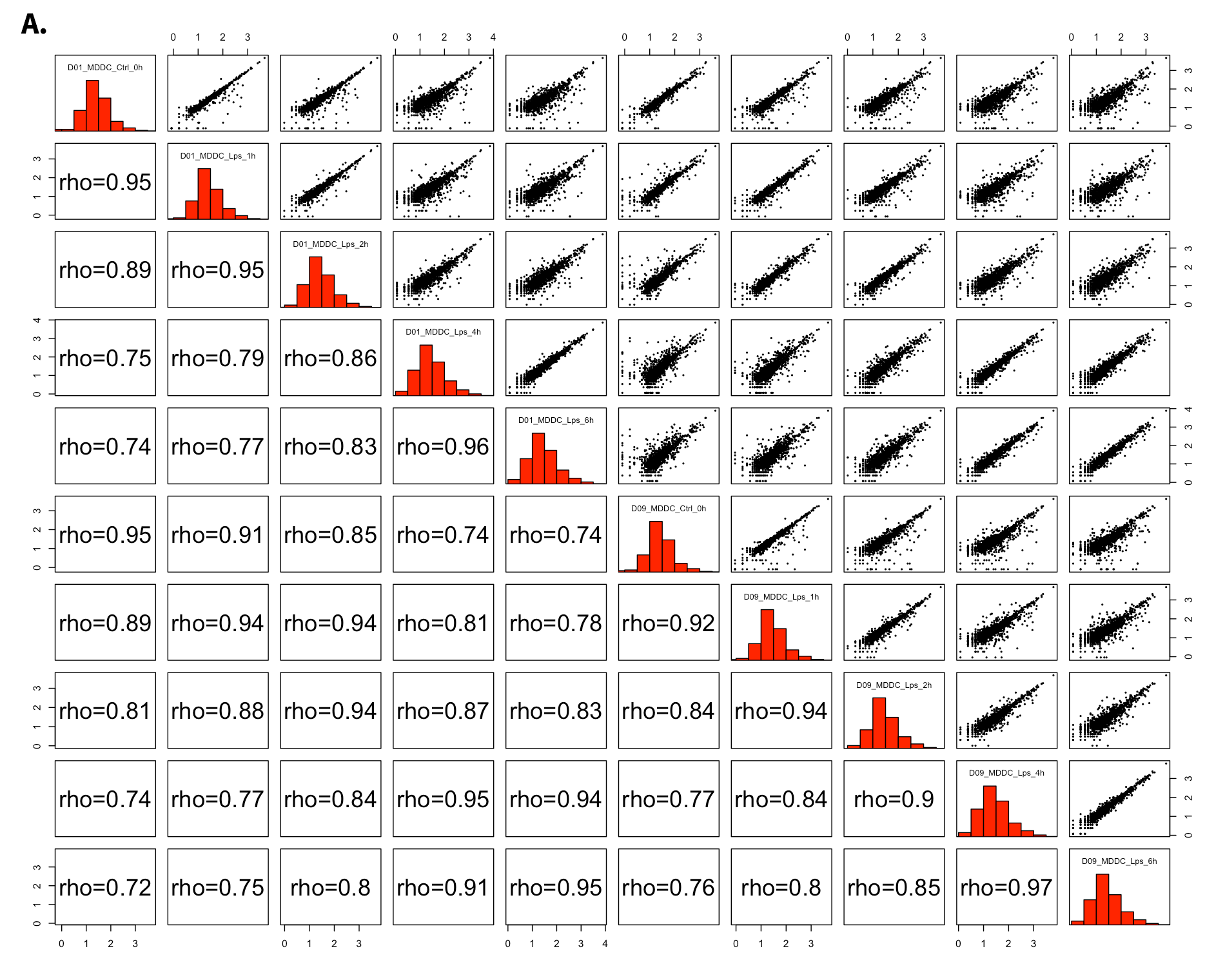


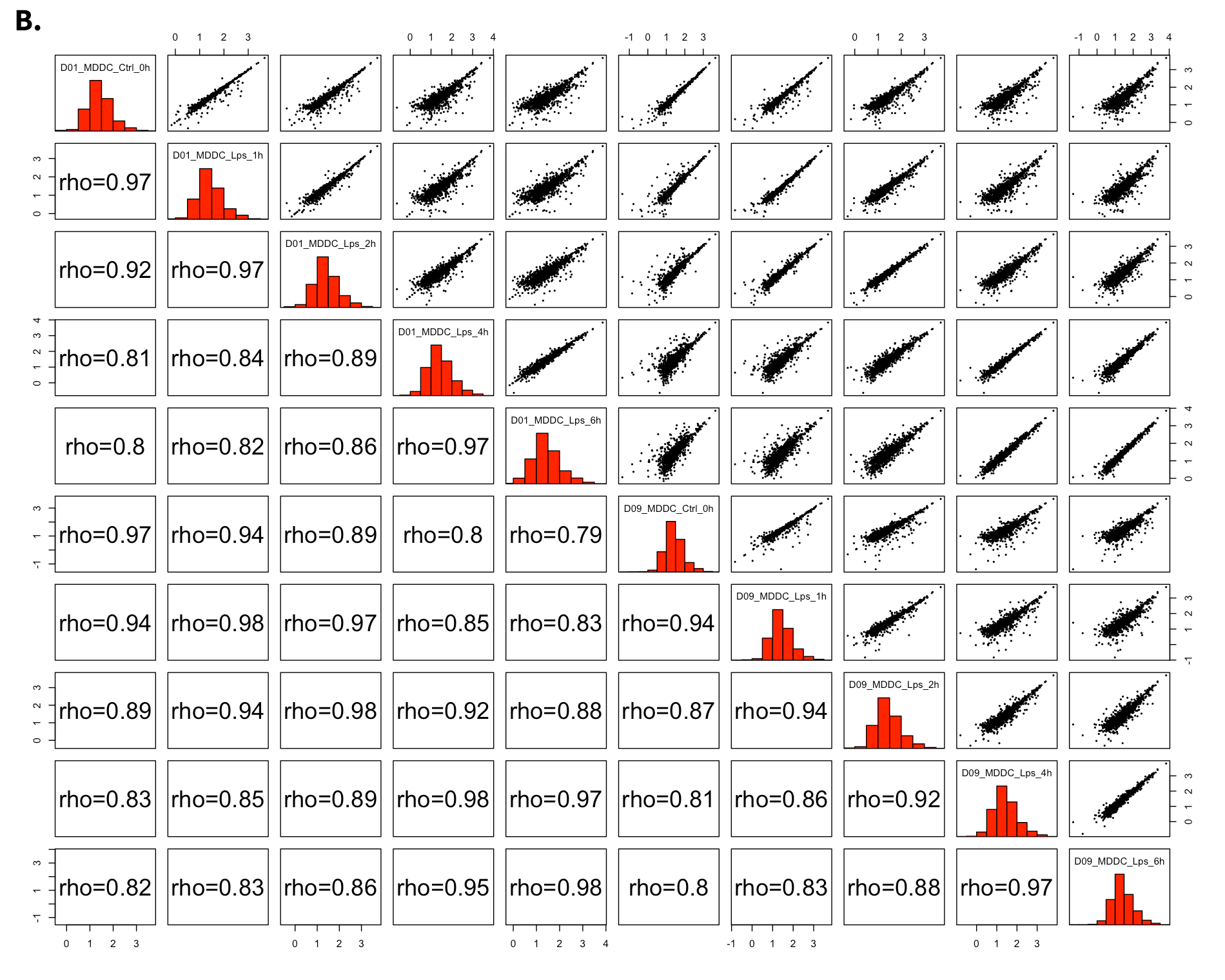


**Figure S2.** All2all scatter plot before (A) and after (B) batch correction. The upper side of the plot displays log10 normalized scatter plots. Bottom side of the plots are the pearson correlations of corresponding samples. Diagonal shows the histogram of normalized read counts in log10 scale.


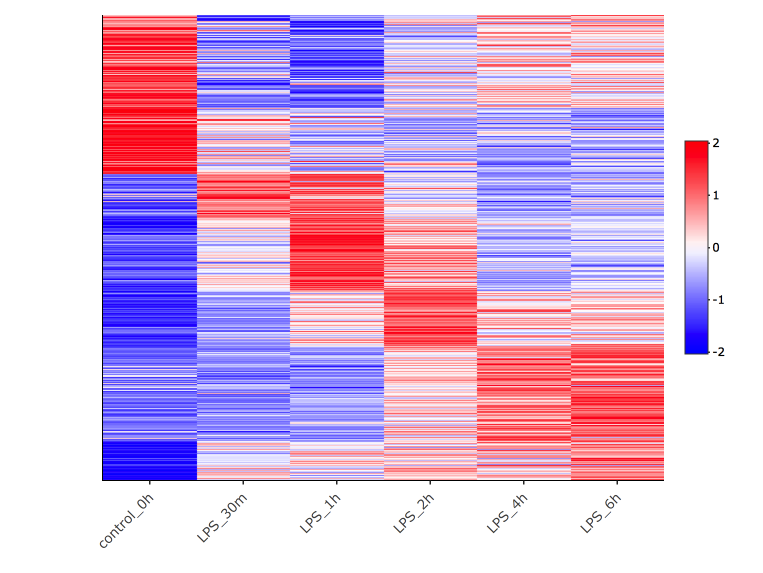


**Figure S3.** Heatmap of open chromatin regions affected by LPS stimulation. The heatmap data is scaled and centered Log_10_ transformed of normalized read counts


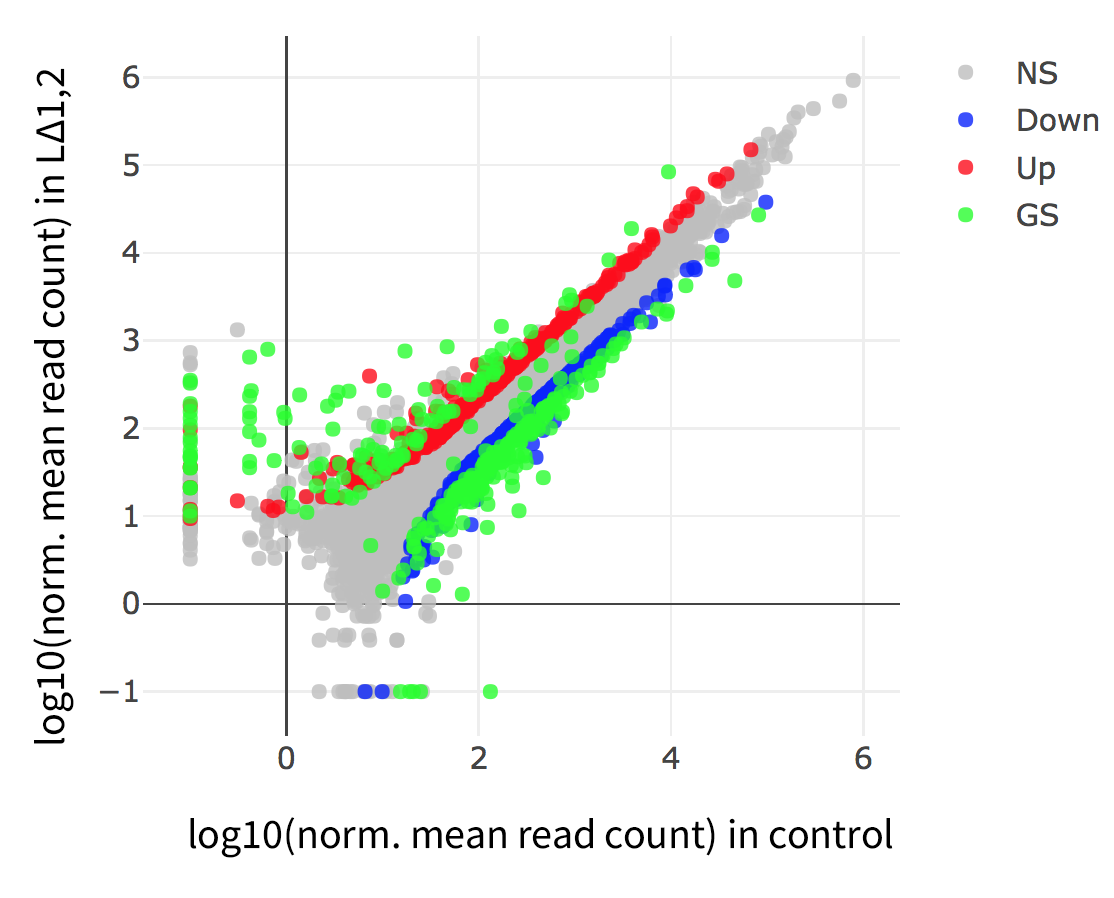


**Figure S4.** Comparison of DE genes which are published by Vernia S. et al. 2014, Fig. S2a (are shown as green circles) and detected genes by DEBrowser (The genes with |log2foldChange|>1 and padj<0.01 are shown as red and blue circles). Normalized count data of the average of all three replicates for Jnk1, Jnk2 double knock out vs. control is shown in log10 scale.


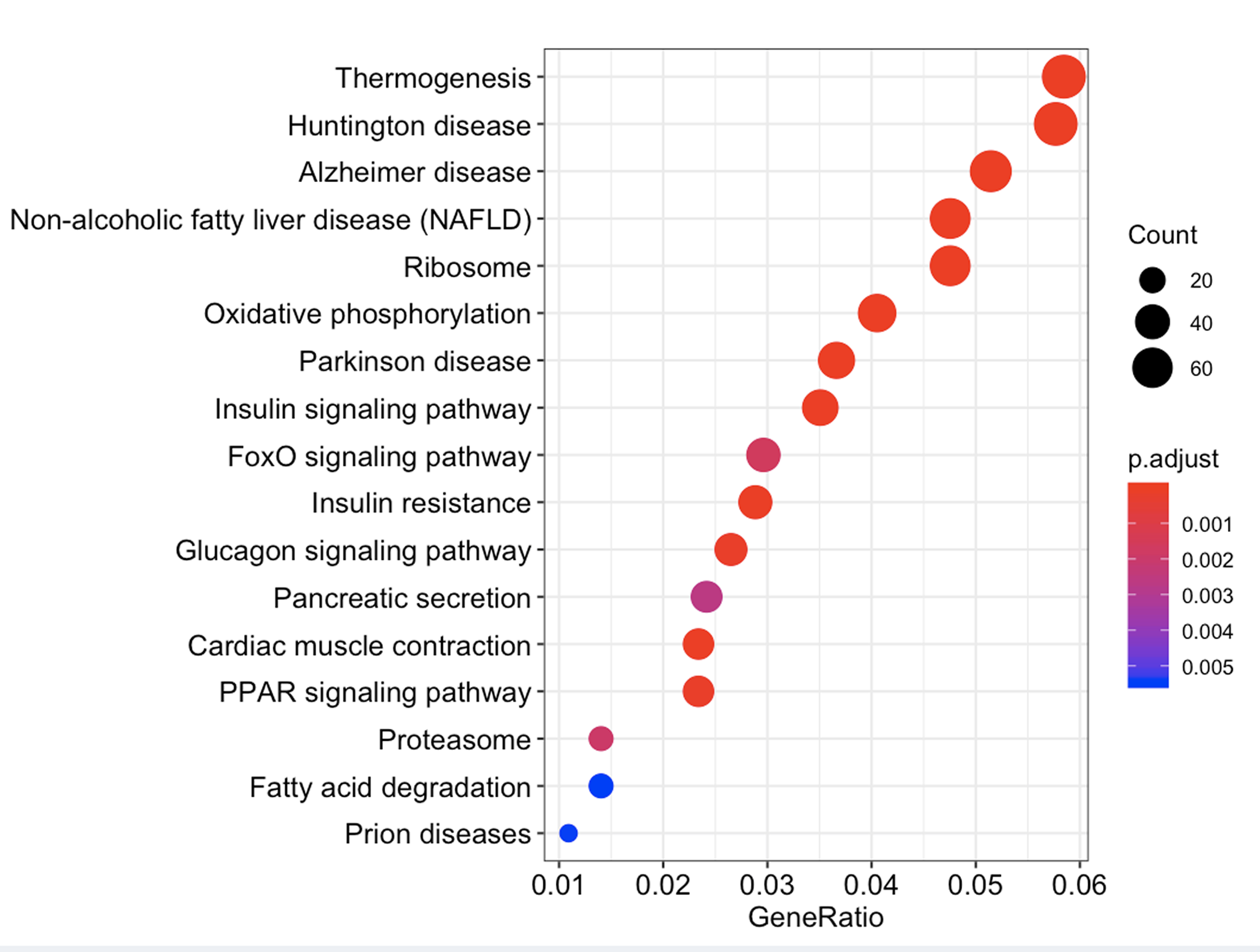


**Figure S5.** Enriched KEGG categories (p_adj_ < 0.01) detected by DEBrowser captures all reported categories in Verina et. al 2014, Fig S3. H.


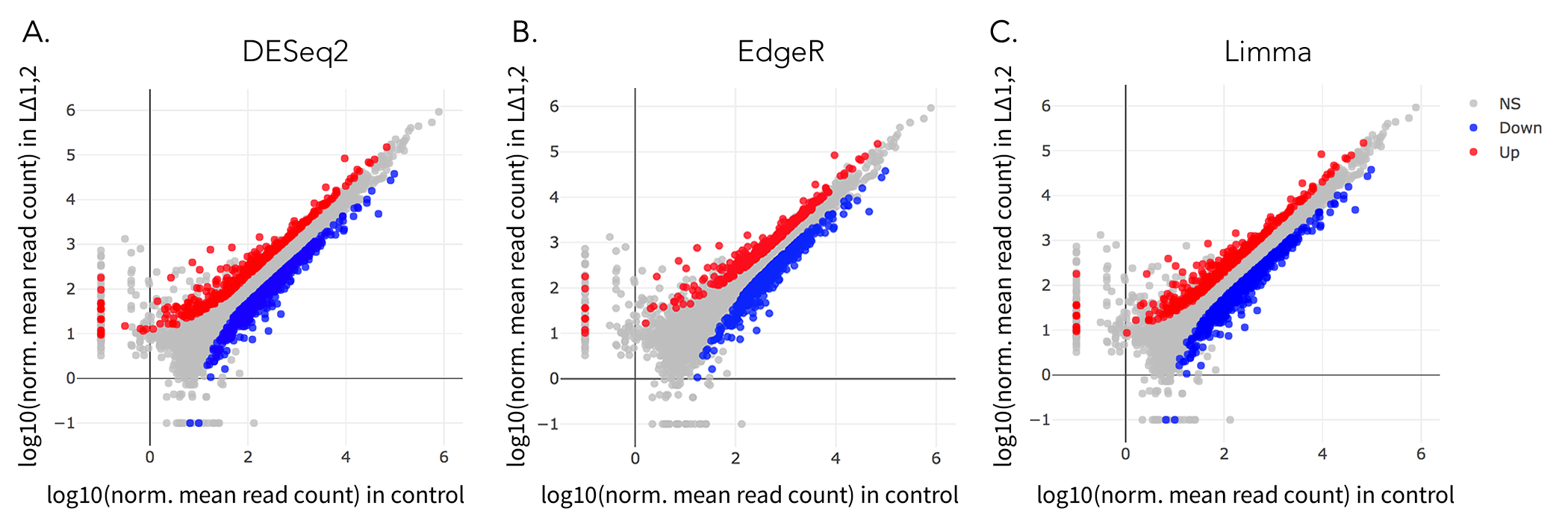


**Figure S6.** Comparison of **A.** DESeq2**, B.** EdgeR and **C.** Limma on data that published by Vernia S. et al. 2014. Normalized count data of the average of all three replicates for Jnk1, Jnk2 double knock out vs. control is displayed in log10 scale. (The cut-offs: |log2foldChange|>1, padj<0.01)

**
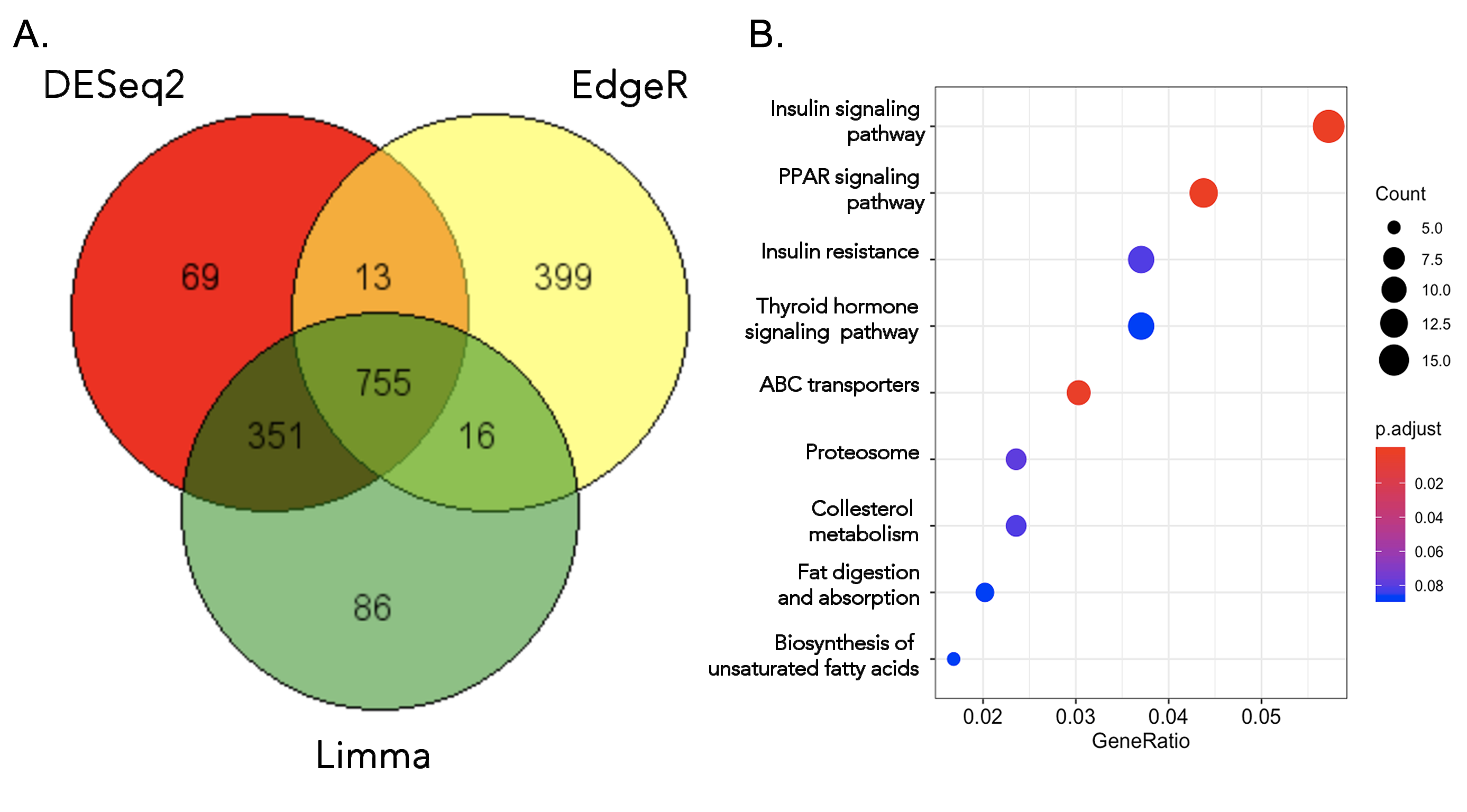
Figure S7.** **A.** Wenn diagram of comparisons between DESeq2, EdgeR and Limma on data that published by Vernia S. et al. 2014. **B.** GO term analysis performed using the intersection in the Venn diagram. Found pathways using these 755 genes are more relavent to the Jnk1 and Jnk2 functions discussed in the Vernia S. et al. 2014. Using the intersection helped eliminating the noise.


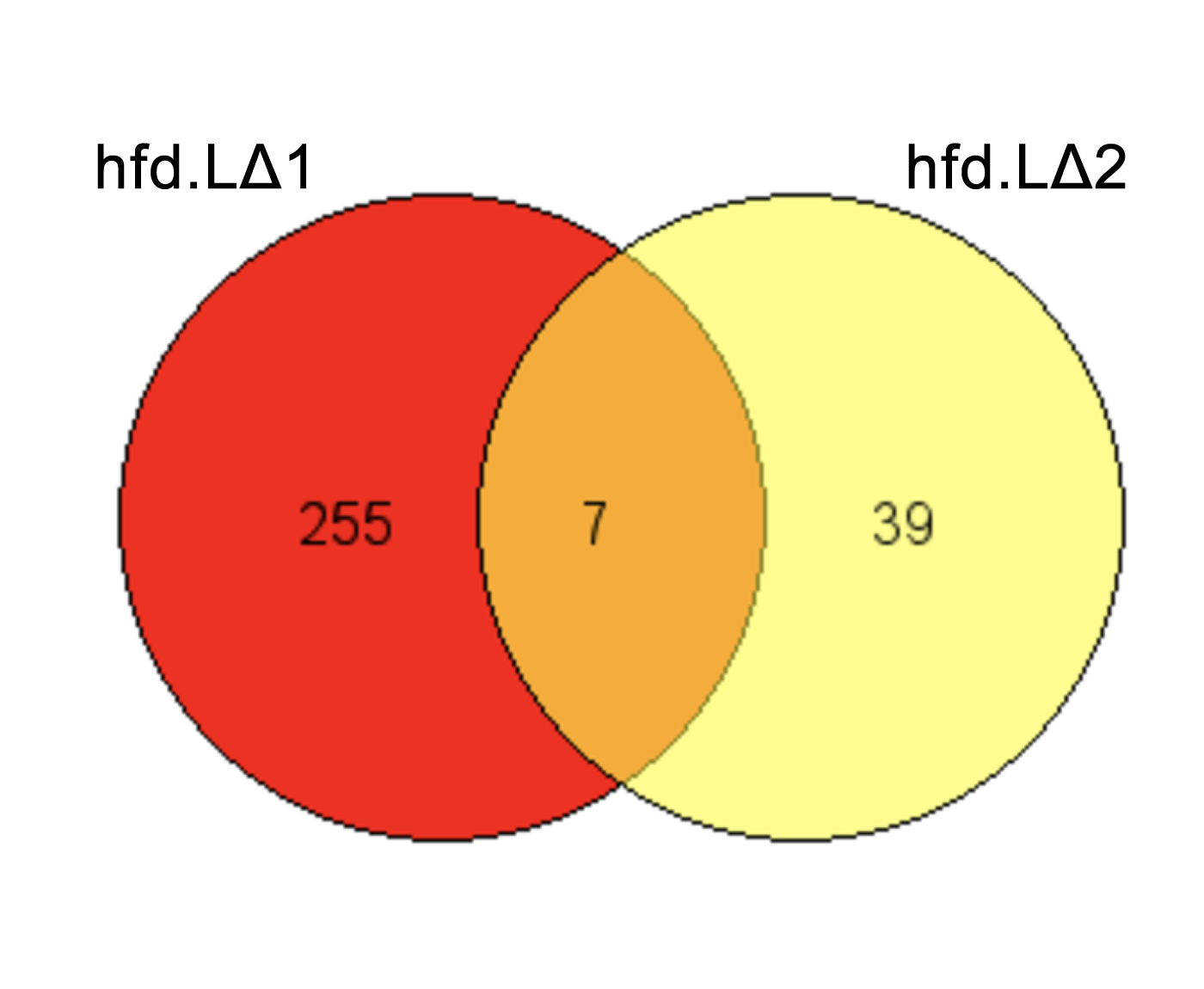


**Figure S8.** Comparison of DE analysis results for hfd.L^Δ1^ vs hfd.WT and hfd.L^Δ2^ vs hfd.WT. High fat diet has a stronger effect on L^Δ2^  animals compared L^Δ1^ animals.
