# Supplementary material for: DEBrowser: interactive differential expression analysis and visualization tool for count data
Source: BMC Genomics. 2019 Jan 5;20:6. doi: 10.1186/s12864-018-5362-x (PMC6321710; doi:10.1186/s12864-018-5362-x)
Supplement: Supplementary file 3 — Data processing methods and installation instructions. (DOCX 18 kb) [file 12864_2018_5362_MOESM3_ESM.docx]

# Data processing methods:

DEBrowser requires a count matrix as input. Each sequencing method requires different processing steps. In this document the users can find the steps taken to process the raw sequencing data into a count matrix. This count matrix can then be uploaded to DEBrowser to perform differential analysis.

**RNA-Seq:**

We first performed a quality filtering step to remove and trim the reads that had lower qualities using Trimmomatic-0.32 [[1]](https://paperpile.com/c/39y50S/N7mv). We trimmed 5nt of 5’ and 3’ ends of bases having an average quality of less than 20 and removed the reads when the average quality is less than 20 in a window size of 10. We also removed any read less than 36 bases. Ribosomal RNA reads were then filtered out using Bowtie2 v2.2.3 [[2]](https://paperpile.com/c/39y50S/mKes) with parameters -p 2 -N 1 –no-unal. RSEM v1.2.29 [[3]](https://paperpile.com/c/39y50S/D4tL) was used to estimate gene counts, with parameters -p 4 –bowtie-e 70 –bowtie-chunkmbs 100 –strand-specific. RSEM is used with Bowtie v0.12.9. Annotation files were downloaded from UCSC Genome Browser [[4]](https://paperpile.com/c/39y50S/CUst); hg19 for human, and mm10 for mouse were used. RSEM results from each sample were then merged to create the count matrix that was uploaded to DEBrowser. Specifically, a tutorial about RNA-Seq data processing and the scripts we used are in a github repository below.

<https://github.com/UMMS-Biocore/RNASeqTutorial>

**ATAC-Seq:**

First, the adapter sequences were removed with Cutadapt version 1.3 [[5]](https://paperpile.com/c/39y50S/SIZu), Trimmomatic was used to remove low quality reads[[1]](https://paperpile.com/c/39y50S/N7mv), and Bowtie2 v2.2.3 [[2]](https://paperpile.com/c/39y50S/mKes), parameter –X 2000 was used for paired end alignments. Reference genome hg19 for human was used. Picard’s MarkDuplicates version 1.131 with Samtools 0.1.19 [[6]](https://paperpile.com/c/39y50S/rp6E) were used to remove PCR duplicates. To accurately call to peaks in the Tn5 transposase cut sites, each mapped read location first shifted 9-bases to the upstream region, where the Tn5 transposase cuts the DNA, and then the reads were extended 29-bases downstream for smoothing. Peaks were then called using MACS2 [[7]](https://paperpile.com/c/39y50S/g7m3) with these adjusted 29-base aligned reads with parameters –bw 29 –tsize 29. We then merged peaks from different samples using Bedtools 2.27.0 merge function to prepare consensus peak locations from each sample. The number of reads in each peak location was quantified using the Bedtools 2.27.0 [[8]](https://paperpile.com/c/39y50S/dmyq)  coverage function. The results were then merged to a single count matrix to perform downstream analysis in DEBrowser.

**ChIP-Seq:**

Paired-end reads were removed when the average quality scores in sliding window size 10 were less then 15, and trimmed when leading and trailing bases with quality scores less than 15 using trimmomatic version 0.32 [[1]](https://paperpile.com/c/39y50S/N7mv). Reads that were longer than 25 bases after trimming were kept for further analysis. The reads were then aligned to human reference genome hg19 using Bowtie2 v2.2.3 [[2]](https://paperpile.com/c/39y50S/mKes) with options --un-conc to filter out reads that align un-concordantly. Duplicated reads were filtered out using Picard’s MarkDuplicates version 1.131. Peaks were then called using MACS2 [[7]](https://paperpile.com/c/39y50S/g7m3). Alignment files were also converted to tdf format using IGVtools count function version 2.3.31 using -w 5 parameter. The called peaks from different samples merged to get consensus peak regions in all samples to create a count table using Bedtools 2.27.0 [[8]](https://paperpile.com/c/39y50S/dmyq) merge function. The number of reads in each consensus peak was quantified with Bedtools 2.27.0 coverage function. The results merged to a count table to perform downstream analysis in DEBrowser.

**Complex Figures:**

**Figure 6B:** To generate this figure, we first searched the genes in insulin pathway using search box in data options menu. The search option colors searched genes on the scatter plot in green. The legend of this plot is also interactive and NS, up and down regulated genes can be removed or isolated within the plot by clicking on each legend. Now, it is easy to select these genes using plotly’s box or lasso select function. When these genes are selected, a heatmap is displayed on the right side of this panel.

**Figure 6C:** To create this plot, first, all pairwise comparisons need to be performed by choosing them in condition selection page. After all DE runs finished, click the “Go main plots” button, and then the “Tables” tab is clicked. Here the dataset needs to be chosen as the “comparisons” in the “Dataset Options” section. In this way, the user can monitor comparison results as a single table. To create a heatmap, QC plots tab, plot type heatmap option is used.

**Figure 6D:** The data first prepared prior to upload it to DEBrowser using excel. In this specific case, we first calculated the means of the triplicates for chow.wt, chow.dbl, hfd.wt and hfd.dbl. We then divided the normalized counts to chow.wt for the genes in PPARα pathway. We then uploaded this comma separated file to DEBrowser to visualize it as a heatmap.

# References:

[1. Bolger AM, Lohse M, Usadel B. Trimmomatic: a flexible trimmer for Illumina sequence data. Bioinformatics. 2014;30:2114–20.](http://paperpile.com/b/39y50S/N7mv)

[2. Langmead B, Salzberg SL. Fast gapped-read alignment with Bowtie 2. Nat Methods. 2012;9:357–9.](http://paperpile.com/b/39y50S/mKes)

[3. Li B, Dewey CN. RSEM: accurate transcript quantification from RNA-Seq data with or without a reference genome. BMC Bioinformatics. 2011;12:323.](http://paperpile.com/b/39y50S/D4tL)

[4. Karolchik D, Barber GP, Casper J, Clawson H, Cline MS, Diekhans M, et al. The UCSC Genome Browser database: 2014 update. Nucleic Acids Res. 2014;42 Database issue:D764–70.](http://paperpile.com/b/39y50S/CUst)

[5. Martin M. Cutadapt removes adapter sequences from high-throughput sequencing reads. EMBnet.journal. 2011;17:10–2.](http://paperpile.com/b/39y50S/SIZu)

[6. Li H, Handsaker B, Wysoker A, Fennell T, Ruan J, Homer N, et al. The Sequence Alignment/Map format and SAMtools. Bioinformatics. 2009;25:2078–9.](http://paperpile.com/b/39y50S/rp6E)

[7. Zhang Y, Liu T, Meyer CA, Eeckhoute J, Johnson DS, Bernstein BE, et al. Model-based analysis of ChIP-Seq (MACS). Genome Biol. 2008;9:R137.](http://paperpile.com/b/39y50S/g7m3)

[8. Quinlan AR, Hall IM. BEDTools: a flexible suite of utilities for comparing genomic features. Bioinformatics. 2010;26:841–2.](http://paperpile.com/b/39y50S/dmyq)
